# Dilution effect of the building area on energy intensity in urban residential buildings

**DOI:** 10.1038/s41467-019-12852-9

**Published:** 2019-10-30

**Authors:** Jingxin Gao, Xiaoyang Zhong, Weiguang Cai, Hong Ren, Tengfei Huo, Xia Wang, Zhifu Mi

**Affiliations:** 1 Chongqing University School of Management Science and Real Estate, Chongqing University, Chongqing, PR China; 2 Institute of Environmental Sciences (CML), Leiden University, Einsteinweg, 2, 2333 CC Leiden, The Netherlands; 3 School of Economics and Management, Hebei University of Technology, Tianjin, 300401 PR China; 4 School of Public Finance and Taxation, Southwestern University of Finance and Economics, Chengdu, PR China; 5 The Bartlett School of Construction and Project Management, University College, London, London WC1E 7HB UK

**Keywords:** Energy and society

## Abstract

Urban residential buildings make large contributions to energy consumption. Energy consumption per square meter is most widely used to measure energy efficiency in urban residential buildings. This study aims to explore whether it is an appropriate indicator. An extended STIRPAT model was used based on the survey data from 867 households. Here we present that building area per household has a dilution effect on energy consumption per square meter. Neglecting this dilution effect leads to a significant overestimation of the effectiveness of building energy savings standards. Further analysis suggests that the peak of energy consumption per square meter in China’s urban residential buildings occurred in 2012 when accounting for the dilution effect, which is 11 years later than it would have occurred without considering the dilution effect. Overall, overlooking the dilution effect may lead to misleading judgments of crucial energy-saving policy tools, as well as the ongoing trend of residential energy consumption in China.

## Abstract (web summary)

Improving residential building energy consumption (RBEC) efficiency is important. The authors here test whether the measure truly reflects efficiency, and found that RBEC per square meter decreases with the increase of the building area per household, indicating the dilution effect.

## Introduction

Human activities are influencing the process of global climate change by emitting large amounts of greenhouse gases into the air, and global warming has become the most significant environmental problem facing humankind so far^1,2^. Approximately one-third of global greenhouse gas emissions and 40% of energy consumption is associated with the building sector^3^. As the largest carbon emitter, China accounts for over one-quarter of the world’s total carbon emissions, and the global carbon reduction trend is highly correlated with China’s emission reduction^4^. Although China’s economic growth has entered a new normal stage, the urbanization process will undoubtedly continue after decades of vigorous development^5^. As a result, the number and scale of Chinese cities have increased dramatically, encouraging the rapid expansion of buildings and its attendant surging energy consumption. The urban residential building energy consumption (URBEC) has increased from 309 Million ton coal equivalent (Mtce) in 2001 to 857 Mtce in 2015 in China. At the same time, with the deepening of the industrialization process, industrial energy savings potential has been declining. Therefore, the building sector has to assume more energy-saving and emission-reduction tasks. Currently, China’s building energy consumption mainly comes from industrial buildings, public buildings and residential buildings^6^. Although the energy consumption per square meter for urban residential buildings (URBEC) is much lower than that of public buildings, the total floor area of residential buildings in China occupies a very high proportion of the total construction area. In addition, the demand for energy consumption caused by the improvement of housing conditions is also increasing with the continuous improvement of people’s living standards^7^. Therefore, the residential sector has a very large potential for decreasing energy consumption and environmental impact.

Improving the residential building energy efficiency plays an essential role in reducing the URBEC. Therefore, it is of great importance to measure energy efficiency effectively, which may facilitate energy conversation policy-making. Two methods are being applied extensively for the URBEC efficiency evaluation. The first method is to directly compare the URBEC of similar buildings at a project level. The other method is to employ the URBEC per square meter as the indicator of energy efficiency, an approach which is mainly used at the industry level. Due to its ease of use and relatively low cost, the URBEC per square meter has been widely applied in most studies, research reports, energy efficiency design standards, and other relevant documents issued by universities, scientific research institutes and governments^8–10^.

Previous literature took the URBEC per square meter as an important efficiency indicator to analyze the driving factors of energy consumption^11–19^, measure energy use performance^11,14,20^, and evaluate the effect of policy tools and make projections^16,18,21^. China Building Energy Use, a widely used annual report published by the Building Energy Research Center of Tsinghua University, explores the energy use status, energy-saving potential, as well as sustainable development paths in the building sector in China, taking the energy use per square meter as an efficiency index^22^. The national building energy saving design standard (BESD) for residential buildings and a number of local BESDs also adopt energy consumption per square meter as the criterion for judging the energy use performance of building operation^23–26^. In addition, the American Council for an Energy Efficient Economy (ACEEE) also evaluates the energy conservation achievements of the building sector in global regions (including China) with the indicator kilojoules per square meter.

These studies, reports, and standards play important roles in facilitating government policy-making and efforts to upgrade building performance, which directly affect the progress of energy conservation and environmental protection. Therefore, whether it is reasonable to use the URBEC per square meter as the benchmark indicator for changing energy use efficiency is critical to energy-saving work and conducting further studies. However, to date, little or no attention has been placed on the rationality of using URBEC per square meter to measure the change in building energy consumption performance.

Here, we propose the dilution effect of building area per household on URBEC per square meter. Data from 867 Chinese households were collected to validate the dilution effect based on an extended STIRPAT model we developed. In brief, the growing floor area per household reduced the URBEC per square meter because of rapid urban sprawl. In other words, the decreasing URBEC per square meter fails to represent the real change in energy use efficiency of building operation without accounting for the dilution effect. We then introduced the interaction term of the BESD and the building area per household into the model. The empirical results show that overlooking the dilution effect leads to significant overvaluation of the effectiveness of implementing BESD in residential buildings, especially those in rapidly urbanized areas. Further analysis finds that the inflection point in the China URBEC is delayed from 2001 to 2012 if the expansion of building area per household is taken into consideration. The full details of models and data sources are available in the Methods section. In addition, we also discuss why the dilution effect phenomenon happens in China, but not everywhere, and how conditions can vary among different economies.

## Results

### The dilution effect of the building area per household

To untangle the impacts of the expansion of the building area per household on the URBEC per square meter, a multivariate linear regression (MLR) model was employed. Based on the survey data, the coefficients of all variables except the Building Floor number passed the 10% significance test.

**Fig. 1:**
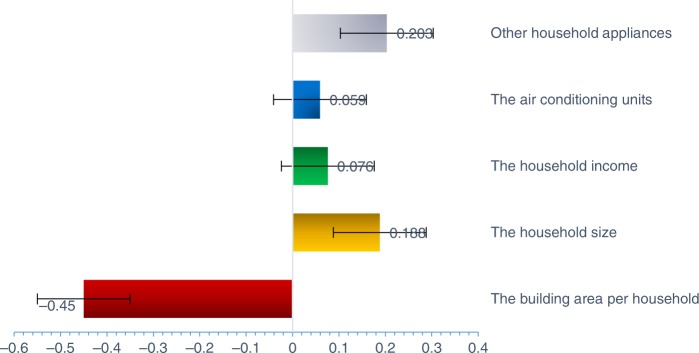
The influence coefficients for the variables. The influence coefficients for household size, household income, number of air conditioning units and other household appliances on the URBEC per square meter are shown in different colors. Error bars are added in excel to donate S.D. (std. deviation)

As shown in Fig. 1, the influence coefficients of the variables Household size (*x*_3_), Household income (*x*_4_), Number of air conditioning units (*x*_5_), and Number of other household appliances (*x*_6_) are positive, indicating that the URBEC per square meter grows in step with the household size, household income, number of air conditionings, and other household appliances. In contrast, the building floor area per household (*x*_1_) is the only variable negatively affecting the URBEC per square meter, and its influence coefficient is −0.450. Thus, the URBEC per square meter will decline as the building area per household increases. In other words, the URBEC per square meter is diluted by the building area per household. Therefore, the survey data in this research validates the dilution effect.

The URBEC per household (*y*_2_) is the product of the URBEC per square meter (*y*_1_) and the building area per household (*x*_1_), as shown in Eq. (1).

1 $${\mathrm{The}}\,{\mathrm{URBEC}}\,{\mathrm{per}}\,{\mathrm{household}}(y_2)\, {\mathrm{ = the}}\,{\mathrm{URBEC}}\,{\mathrm{per}}\,{\mathrm{square}}\,{\mathrm{meter}}(y_1) \qquad \\ \times {\mathrm{the}}\,{\mathrm{building}}\,{\mathrm{area}}\,{\mathrm{per}}\,{\mathrm{household}}(x_1)$$

To intuitively understand how strongly the URBEC per square meter is influenced by the increase of the building area per household, we further explored the impact of the building area per household on the URBEC per household. The results show that the influence coefficient of the building area per household on the URBEC per household is positive (0.133). This finding indicates that the URBEC per household increases with the expansion of the building area per household when other factors are ruled out. Based on Eq. (1), this means that when the building area per household increases and the URBEC per square meter decreases, their product also increases. In other words, the decrease rate of the URBEC per square meter drops in the interval from 0 to the increase rate of the building area per household. The extent of this decrease depends on the gap between the rates of increase of living space and energy consumption per household (see below for further discussion). In regions where the building area per household is expanding rapidly, the dilution effect can be very significant and thus should be carefully considered.

### The overestimation of the BESD effectiveness

As a widely applied policy tool, the building energy saving standard (BESD) plays a crucial role in improving the energy saving performance of urban buildings in China. However, to date, the evaluation of the BESD effectiveness has generally omitted any discussion of being influenced by the rapid expansion of building area per household. To explore how the effectiveness of BESD differs when the dilution effect is accounted for or not, indicating the impacts of the fast-growing building area per household, the interaction term of building area per household (*x*_1_) and BESD is introduced into the MLR model.

**Fig. 2:**
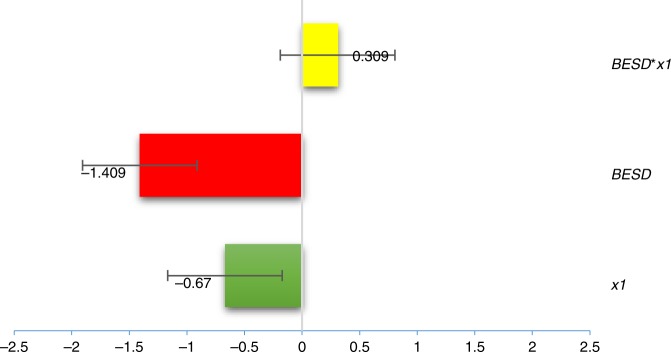
The dilution effect on the building energy saving standard. The influence coefficients of BESD, the building area per household and their interaction term (BESD*x_1_) on the URBEC per square meter

The results show that the influence coefficients of BESD and building area per household on the URBEC per square meter are −1.409 and −0.67 respectively, reflecting that both factors have a positive effect on reducing energy intensity (Fig. 2). However, the coefficient of the interaction term of BESD and building area per square meter is 0.309, which shows a negative effect opposite to their single influence. With this interaction term introduced into the model, the effect coefficients of BESD and building area per square meter decreased by 21.9% and 46.1% to −1.1 and −0.361, respectively. This finding indicates that when the increase of building area per square meter is considered, the degree of reduction in the URBEC per square meter attributed to BESD implementation decreases significantly. For those regions where floor area per household is expanding rapidly, the effectiveness of BESD adoption on energy savings should be reevaluated properly to account for the dilution effect.

### The BESD moderates the dilution effect

**Fig. 3:**
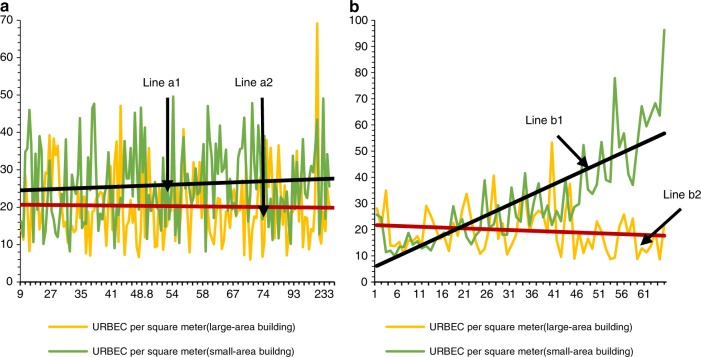
Comparison of the energy intensity between small and large residential buildings. All buildings in group (**a**) have adopted the BESD, and all the buildings in group (**b**) have not adopted the BESD. Lines a1 and b1 are the trend lines of the URBEC per square meter for small residential buildings. Lines a2 and b2 are the trend lines of the URBEC per square meter for large residential buildings. The *x*-axis of figures **a** and **b** represents the difference in the building area (*D*-value) between the matched pairs. The y-axis of figures **a** and **b** represents the URBEC per square meter of the matched pairs

The results also show that the adoption of the BESD in buildings can weaken the dilution effect caused by the increase in building area per household. Figure 3 demonstrates the differences in the URBEC per square meter between two groups of buildings. Buildings in group (a) adopted the BESD, while buildings in group (b) did not adopt the BESD. In both cases, the gap between the energy intensity of large-area buildings and smaller ones is obvious, which is consistent with the dilution effect. However, for buildings that meet the BESD, the reduction of the URBEC per square meter aroused by the expansion of floor area per household is less noticeable. In fact, the BESD is better implemented in more developed regions, where the pursuit of higher indoor comfort level eases the gap between the growth of the URBEC and building area per household. In general, the dilution effect could be more significant in emerging economies.

### Delayed inflection point of the China URBEC

The inflection point of the URBEC per square meter is widely considered an important indicator to predict the evolution trend and the temporal peak of the URBEC in China^27^. As indicated by the IPAT model and Kuznets curve theory, the occurrence of the inflection point of energy intensity signifies that the urban population, economic growth, and technological level have reached a certain level, at which point further development would lead to a reduction in the increment in the energy consumption.

**Fig. 4:**
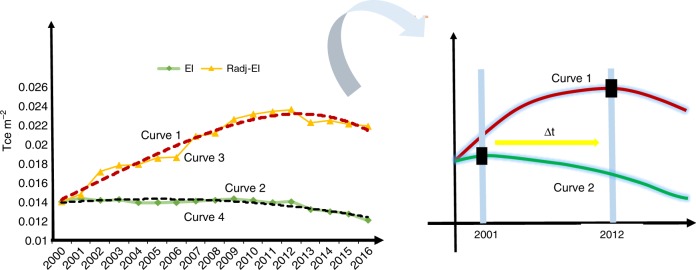
Change trend and inflection point of the energy intensity. **a** URBEC per square meter from 2000 to 2016 in China. **b** The inflection point of the URBEC per square meter. Curve 1 refers to the trend line of the URBEC per square meter without considering the dilution effect. Curve 2 refers to the trend line of the URBEC per square meter considering the dilution effect. Curve 3 refers to the line chart of the energy intensity (URBEC per square meter) considering the dilution effect. Curve 4 refers to the line chart of the energy intensity (URBEC per square meter) without considering the dilution effect. Δt is the delayed time between the inflection points of curve 1 and curve 2. EI represents the URBEC per square meter without considering the dilution effect and Radj-EI represents the URBEC per square meter with considering the dilution effect

Here, we estimated the inflection point of the URBEC per square meter of China with and without considering the dilution effect. Figure 4 shows the change patterns of the URBEC per square meter in China from 2000 to 2016. The inflection point of URBEC intensity occurred in 2001 without considering the dilution effect. If the dilution effect is taken into account, the inflection point occurred in 2012, 11 years later than it would have occurred if the dilution effect was not considered. Thus, from 2001 to 2012, the energy intensity was still increasing (rather than decreasing) as the growth of the population, the economy, and technology increased. Only after 2012 has the URBEC per square meter truly reversed its rising trend. Since China is still undergoing a rapid expansion of urban building area, neglecting the dilution effect may cause misleading judgments about the ongoing trend of the URBEC in China.

## Discussion

The past decades have witnessed a dramatic increase in the living space of urban residents, and this growth trend is expected to continue in the future. As a result, the building area per household has also been increasing, especially in many emerging economies. In the meantime, economic development contributes to the pursuit of better indoor comfort levels and living conditions in urban areas, thus leading to a higher energy demand per household. Against such a background, energy efficiency improvement is important to save energy resources and reduce the environmental impact.

The URBEC per square meter is widely considered as a benchmark indicator of energy intensity to measure energy use performance, estimate the effectiveness of policy tools, and understand trends. While it is obvious that the energy use per household directly affects the energy intensity, the impacts of growing building area per household on the URBEC per square meter have been overlooked. Here, we analyze the socioeconomic background of the dilution effect in China, as well as how this differs from other economies. In general, how the URBEC per square meter varies with increasing floor area per household depends on the gap between the growth rates of building area and energy use per household.

Energy consumption of residential buildings mainly result from the operation of household appliances (including heating, air conditioning, and other appliances)^28^. The number and the use intensity of these appliances are closely related to the number of residents and the building area^29^. According to the design standard of residential buildings in China, the volume of energy consumption of one building can be expressed as: E_*t*_ = *e*_1_ ∗ *A* + *e*_2_ ∗ *N*. E_*t*_ represents the energy consumption of the entire building at time *t*. *e*_1_ is the energy consumption per square meter consumed by the appliances (heating, for example) associated with the floor area. *e*_2_ is the energy consumption per capita consumed by the appliances (cooking, for example) associated with the number of residents. A denotes the total living area of the entire building. *N* is the number of the residents living in the building. Therefore, the energy consumption per square meter of the entire building can be given as:

2 $$e_t = \frac{{e_1 \ast A + e_2 \ast N}}{A} = e_1 + e_2 \ast N/A = e_1 + e_2/(A/N)$$

where *e*_*t*_ represents the URBEC per square meter. In modern China, the household size has steadily declined since 1982^30^ and then gradually leveled off in recent years^31^. Meanwhile, there are no obvious differences in household size from family to family. In 2015, the State Health Planning Commission released China’s Household Development Report (2015). The report shows that the household size has been decreasing, and families of two and three members has become the mainstream type^32^. By contrast, the building area per household has shown an increasing trend since 1950. Therefore, this paper proposes two hypotheses: first, the household size has changed little in recent years; second, the building area per household has been increasing continuously^33^. Under the first hypothesis, the building area per capita can be expressed by formula (3).

3 $$A/N = a/n$$

*a* is the building area per household, and n is the household size. The energy consumption per square meter can be expressed by formula (4).

4 $$e_t = e_1 + e_2/(a/n)$$

For most regions, *e*_1_ and *e*_2_ are increasing at certain speeds depending on a number of economic, social, and environmental factors. With both the energy consumption (*e*_1_ and *e*_2_) and the building area per household (*a*) growing, the change pattern of *e*_*t*_ can vary significantly. In rapidly urbanized areas of China (Chongqing for example), the floor area per household has increased dramatically, overwhelming the growth pace of energy consumption (*e*_1_, and *e*_2_). This can be attributed to the following reasons. First, an aging population and a migration from rural to urban areas squeezed living space for urban residents, leading to a rigid housing demand years ago. Second, with economic development, wealthier urban residents have a strong desire for larger living spaces, which has been nurtured by the booming real estate construction in recent years. The National Bureau of Statistics reported that floor area per household in urban China more than quintupled from 6.7 m^2^ in 1978 to 36.6 m^2^ in 2016^34^. Third, although people have been pursing more comfortable indoor environments, leading to higher residential energy consumption demands, the continuous improvement in building energy-saving performance moderates the upward trend of the URBEC per household.

The market-oriented reform of the Chinese real estate industry launched in the 1980s has made remarkable achievements. In the meantime, several problems have also been created in this booming industry. First, the faulty market mechanism in the real estate industry has failed to guide normal investment, resulting in a large number of speculative actions. Second, due to the imbalance between supply and demand, the market demand is huge, while the effective supply is insufficient. Moreover, the housing price-income ratio in some cities is exorbitant, thus boosting the rental market in China. As a result, both multi-house families (families owing more than one house) and renters account for a large proportion of the population in urban China, which is highly correlated with the data collection and findings of our research. In general, the reasons why people purchase more houses than they need are, to avoid summer heat or winter cold, for investment, and for earning rental income.

**Fig. 5:**
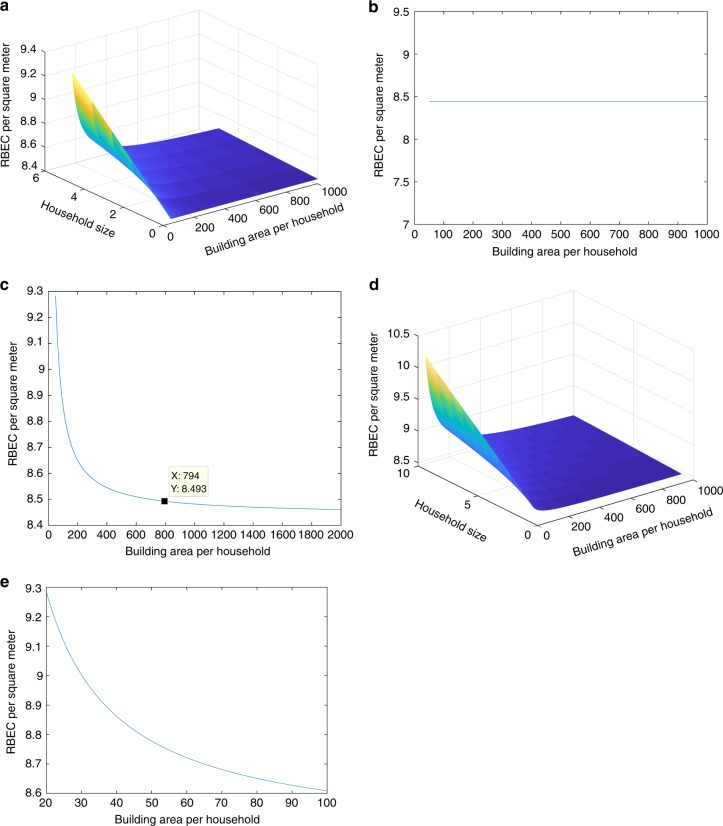
The influence of different phenomena on the dilution effect. **a** Influence of the multi-house family (for avoiding summer heat or winter cold). The residence time for these houses is shortened greatly. According to Eq. (4), this will lead to an amplification (for large houses) or weakening (for small houses) effect on the dilution effect. **b** Influence of the multi-house family (for investment). Many houses will be left vacant, for which *e*_1_ = 0 and *e*_2_ is a constant. This leads to a similar influence on the dilution effect as the first scenario, but the amplification and weakening effects are more pronounced. **c** Influence of the family tenant. In this case, a family lives normally in the house and the dilution effect is not influenced. **d** Influence of the multi-person tenant. In this case, hypotheses (a) and (b) above are invalid. This leads to a weakening (for large houses) or amplification (for small houses) effect on the dilution effect. **e** Influence of the single-person tenant. In this case, *n* = 1. This leads to an amplification (for large houses) or weakening (for small houses) effect on the dilution effect

To illustrate how these different phenomena influence the dilution effect, a scenario analysis is conducted in this paper (see Fig. 5).

To eliminate the influences of these disturbing factors and maintain consistency of the climate zone, customs, living habits and other uncontrollable factors, we followed three criteria to collect the samples which includes that the actual residence time in the house is more than 8 months during one year; the households are owners of the houses or family tenants; and the houses are located in the main urban area of Chongqing.

Overall, our results show that the growing building area per household significantly affects the urban residential energy use per square meter in rapidly urbanized regions. Overlooking the dilution effect may lead to misleading judgments of energy conservation progress, as well as the effectiveness of sustainable policy tools. Under a 2 °C warming target, options to manage residential energy use play increasingly important roles in global carbon emission reductions. Efforts to promote residential building energy use performance must be enhanced to align with the sustainable environmental goals, and whether policy actions are truly effective highly depends upon the rationality of energy use estimation.

## Methods

### Extended STIRPAT model

Accompanied by the progress of industrialization, environmental problems are becoming increasingly severe. American biologists proposed the theory of technical determinism, indicating that the expansion of industrial production is the main cause of environmental problems^35^. Ehrlich et al.^35,36^ argued that the main cause of environmental problems was population growth. In follow-up studies, scholars realized that environmental issues are not shaped by a single factor. Therefore, Ellic and Holton proposed the famous IPAT model:^37^

5 $${I} = {P} \times {A} \times {T}$$

where *I* represents the environmental impact. *P* denotes the population factor. *A* is the affluence level. *T* is the technical level. However, the premise of the IPAT model is that the effects each variable on the dependent variable are the same^38^. To overcome this limitation, Dietz et al^39^. proposed the STIRPAT model.

6 $${I} = {\alpha P}_{it}^{\beta _1}{A}_{it}^{\beta _2}{T}_{it}^{\beta _3}e_{it}$$

where α is a constant. *β*_1_, *β*_2_, and *β*_3_ are the parameters to be estimated, and *e* represents the random error. To address the heteroscedasticity of the model, we took the natural logarithm of *I* in formula (10) to obtain the following equation:

7 $${\mathrm{ln}}I_{it} = \alpha + \beta _1{\mathrm{ln}}P_{it} + \beta _2{\mathrm{ln}}A_{it} + \beta _3{\mathrm{ln}}T_{it} + e_{it}$$

The STIRPAT model is more extensible, and overcomes the limitations of the IPAT model, allowing variables to be added or removed according to research purposes. Considering previous literature (see Supplementary Table 1), the household size (*x*_3_) and the household income (*x*_4_) were selected as indicators of the population (variable *P* in the IPAT model) and affluence (variable *A* in the IPAT model), respectively. However, the technology indicator is an important, but difficult-to-quantify, variable. In this research, the technologies to promote the URBEC efficiency include improvements in the overall heat transfer coefficient of the building envelope, the shape coefficient of the building, the window-to-wall ratio, the heating system and so on. Therefore, the BESD of urban residential buildings, which embodies the performance of these improvement interventions, was selected as the indicator of technology level (variable T in the IPAT model). In addition, the number of floors was introduced into the STIRPAT model as variable *x*_2_, since it significantly impacts lighting and ventilation. The number of the air conditioning units (*x*_5_) was particularly employed as a key variable in this model, as Chongqing is typically hot in summer and cold in winter. Other household appliances were denoted as *x*_6_. Therefore, formulas (8) and (9) were established as follows.

8 $${\mathrm{ln}}y_2 = \alpha _0 + \alpha _1{\mathrm{ln}}x_1 + \alpha _2{\mathrm{ln}}x_2 + \alpha _3{\mathrm{ln}}x_3 + \alpha _4{\mathrm{ln}}x_4 + \alpha _5{\mathrm{ln}}x_5 + \alpha _6{\mathrm{ln}}x_6 + \alpha _7D + e_{it}$$

9 $${\mathrm{ln}}y_2 = \alpha _0 + \alpha _1{\mathrm{ln}}x_1 + \alpha _2{\mathrm{ln}}x_2 + \alpha _3{\mathrm{ln}}x_3 + \alpha _4{\mathrm{ln}}x_4 + \alpha _5{\mathrm{ln}}x_5 + \alpha _6{\mathrm{ln}}x_6 + \alpha _7D + e_{it}$$

A MLR model was then employed for parameter estimation to explore the impact of growing building area per household on the URBEC per square meter.

### Impacts on BESD effectiveness

To explore how the growing building area per square meter influences the evaluation of the effectiveness of adopting BESD in residential buildings, the interaction term of BESD and *x*_1_ (*D* ∗ *x*_1_) was introduced into formula (8). We then obtain formula (10) as follows.

10 $${\mathrm{ln}}y_1 =	 \alpha + \beta _0D + \beta _1{\mathrm{ln}}x_1 + \mu D \ast {\mathrm{ln}}x_1 + \beta _2{\mathrm{ln}}x_2 + \beta _3{\mathrm{ln}}x_3 + \beta _4{\mathrm{ln}}x_4 + \beta _5{\mathrm{ln}}x_5 \\ 	 + \beta _6{\mathrm{ln}}x_6 + e_{it}$$

where *μ* is the coefficient of the interaction term *D*∗*x*_1_. If the results show that *β*_0_ is negative while *μ* is positive, then the effectiveness of adopting BESD has been overestimated with the dilution effect neglected. If both *β*_0_ and *μ* are negative, then the impacts go in the opposite direction. With the growing building area per household considered, the real effect of BESD is represented by *μ* + *β*_0_.

### Impacts of adopting the BESD on the dilution effect

The PSM (Propensity Score Matching) model, as a rigorous method to overcome selection bias, uses nonexperimental data or observation data to analyze the intervention effect^40–42^. The law of its extrapolation is as follows: if there is no A, what is the result of B? It uses the scores for sample matching and makes comparisons in order to estimate the value of the Average Treatment Effect on the Treated (ATT)^43,44^.

11 $${\mathrm{ATT}} = {\mathrm{E}}\left[ {Y_1 - Y_0|T = 1} \right]$$

12 $${\mathrm{T}} = \left\{ {\begin{array}{*{20}{l}} {1\quad \mathrm{accept}\,\mathrm{the}\,\mathrm{intervention}} \hfill \\ {0\quad \mathrm{not}\,\mathrm{accept}\,\mathrm{the}\,\mathrm{intervention}} \hfill \end{array}} \right.$$

In formula (11) and (12), *Y*_1_ is the experimental group, and *Y*_0_ is the control group. T is selected as the key indicator variable to identify the experimental group and the control group. Here, we take variable *x*_1_ as the key indicator T. Then, the formula (13) is obtained.

13 $${\mathrm{ATT}} = {\mathrm{E}}\left[ {y_2|\left( {x_1 = 1} \right)} \right] - {\mathrm{E}}\left[ {y_2|\left( {x_1 = 0} \right)} \right]$$

Because *x*_1_ is either 0 or 1 in formula (13), we need to dichotomize the variable *x*_1_ from a continuous variable to a 0–1 variable. First, the data were categorized into group (a) (BESD = 1) and group (b) (BESD = 0) according to the value of the variable BESD. Second, the average values of *x*_1_ were calculated in group (a) and group (b), respectively. The variables *x*_1_ less than the average value were set to 0, and those above the average value were set to 1. Then, the dichotomized value of *x*_1_ was stored in the *x*_11_ variable. Finally, the data were paired by the nearest neighbor matching method.

### Impacts on URBEC inflection point estimation

To eliminate the distorted influence of the dilution effect on the URBEC inflection point prediction, we introduce the area correction index (ACI) referring to the principle of the consumer price index (CPI). Given the time period concerned, the urban residential building area per capita in 2000 was selected as the base year. Therefore, the area correction index (ACI) can be expressed as

14 $${\mathrm{ACI}}_i = \frac{{\mathrm{URBAPC}}_i}{{\mathrm{URBAPC}_{2000}}}$$

where URBAPC_2000_ and URBAPC_*i*_ denote the urban residential building area per capital in the year 2000 and year i, respectively. The adjusted URBEC per square meter (AURBECP) is

15 $${\mathrm{AURBECP}}_i = {\mathrm{URBAPC}}_i \ast {\mathrm{ACI}}_i$$

### Variable selection

A number of factors affect the URBEC per square meter, including macroeconomic factors (economy, culture, and society) and microeconomic factors^45,46^. Based on a review of previous literature, the key factors were classified into two types: area-related factors and residents-related factors^47–59^ (Supplementary Table 1).

The value of variable BESD is 1 when the energy efficiency design standard is adopted. Otherwise, its value is 0.

16 $${\mathrm{BESD}} = \left\{ {\begin{array}{*{20}{l}} {1,\mathrm{the}\,\mathrm{energy}\,\mathrm{efficiency}\,\mathrm{design}\,\mathrm{standard}\,\mathrm{is}\,\mathrm{adopted}} \hfill \\ {0,\mathrm{the}\,\mathrm{energy}\,\mathrm{efficiency}\,\mathrm{design}\,\mathrm{standard}\,\mathrm{is}\,\mathrm{notadopted}} \hfill \end{array}} \right.$$

As the major source of final energy consumed by residential building operation in urban China, electricity consumption was taken to be representative of the URBEC^60^. In this research, the URBEC per square meter (*y*_1_) and the URBEC per household (*y*_2_) are defined as two dependent variables.

The design standard for the energy efficiency of residential buildings (BESD) was released to improve buildings’ energy use performance to reduce energy waste and carbon generation. As a major part of energy-saving efforts, the Chongqing Building Energy Conservation Association Green Building Professional Committee, the Chongqing Construction Technology Development Center, and other related departments have jointly developed the design standard for the energy efficiency of residential buildings^61,62^. Here, we select the building area per household (*x*_1_) as the key explanatory variable and the BESD as the auxiliary variable.

### Data sources

Data for this study were collected in three ways. First, the data for the physical characteristics of the residential buildings, including name, age, and other basic information was obtained from the Chongqing Municipal Commission of Urban-Rural Development (CMCURD). Considering accessibility and convenience, multilevel random sampling techniques and the probability proportional to size (PPS) were applied to select optimal samples. Second, the data for variable values, including building area per household, number of floors, household size, household income, number of air conditioning units, and number of other appliances were collected from a survey conducted by the China Association of Building Energy Efficiency (CABEE) in 2016. A team of 40 undergraduates, 10 graduate students and 4 Ph.D. students completed this survey over almost two months. To guarantee the quality of the survey, all the team members were trained for a week in advance. During the survey, all the team members were required to record all the interviewees’ telephone numbers for follow-up confirmation. The selected families met three criteria. First, the household selected had to provide the electricity billing number or the ID of the electric meter for finding the electricity consumption data from the State Grid Chongqing Electric Power Company (SGCEPC). Second, the households used the energy for consumption purposes rather than production purposes. Third, the households had been living in the building for more than one year, so that the incomparability of the energy consumption data caused by seasonal weather changes over the year can be eliminated. Third, the household electricity consumption data were collected from the SGCEPC. Based on the obtained survey data and monthly electricity consumption data for 867 households, the basic statistical characteristics of the variables are shown in Supplementary Table 2.

### Collinearity test

To avoid the result deviation caused by the collinearity of variables, the variance inflation factor (VIF) was calculated. As shown in Supplementary Table 3, the VIF for all variables is between 1.02 and 1.36. The closer the VIF is to 1, the smaller the collinearity of variables will be. Therefore, the results demonstrate that there is no collinearity among the variables selected in this paper.

## Supplementary information

Supplementary Information

Description of Additional Supplementary Files

Supplementary Data 1

Supplementary Data 2

Supplementary Data 3

Supplementary Data 4

Supplementary Data 5

Supplementary Data 6

## Data Availability

The data for the physical characteristics of the residential buildings, the household electricity consumption and variable values, including building area per household, number of floors, household size, household income, number of air conditioning units, and number of other appliances are in Supplementary data 1. The data for the PSM (Propensity Score Matching) model is in Supplementary data 2. The source data for the Fig. 3 is in Supplementary data 3 and 4. The source data for the Fig. 4 is in Supplementary data 5. Supplementary Table 1 is the factors influencing the URBEC per square meter. Supplementary Table 2 is the statistical characteristics of the variables. Supplementary Table 3 is variance inflation factor of variables.

## Code availability

All the computer code generated during this study is available from the corresponding authors and provided in the Supplementary Data 6.
