# Supplementary material for: Dilution effect of the building area on energy intensity in urban residential buildings
Source: Nat Commun. 2019 Oct 30;10:4944. doi: 10.1038/s41467-019-12852-9 (PMC6821746; doi:10.1038/s41467-019-12852-9)
Supplement: Supplementary file 1 — Supplementary Information [file 41467_2019_12852_MOESM1_ESM.docx]

**Supplementary information**

**Manuscript title：***Dilution effect of the building area on energy intensity in urban residential buildings*

**Jingxin et al.**

**Supplementary table 1,**

**Table 1** The factors influencing the RBEC per square meter

| Type | Variable | Indicator |
| --- | --- | --- |
| Area-related factors | $x_{1}$ | Building area per household^50-52^ |
|  | $x_{2}$ | Number of floors^35, 53^ |
|  | $x_{6}$ | Other household appliances^50, 51, 54^ |
|  | BESD | Building energy saving design standard^55-57^ |
| Residents-related factors | $x_{3}$ | Household size^58, 59^ |
|  | $x_{4}$ | Household income^60^ |
|  | $x_{5}$ | Number of the air conditioning units^61, 62^ |

**Supplementary table 2:**

**Table 2** The statistical characteristics of the variables

| Descriptive statistics | Number  of cases | Minimum | Maximum | Average | Standard  Deviation |
| --- | --- | --- | --- | --- | --- |
| $y_{1}$ (URBEC per square) | 867 | 1.795 | 4.661 | 3.045 | 0.471 |
| $y_{2}$ (URBEC per household) | 867 | 6.304 | 9.311 | 7.456 | 0.491 |
| BESD (Building energy saving design standard) | 867 | 0.000 | 1.000 | 0.660 | 0.474 |
| $x_{1}$ (Building area per household) | 867 | 3.296 | 5.799 | 4.457 | 0.362 |
| $x_{2}$ (Floor number) | 867 | 0.000 | 3.497 | 2.268 | 0.898 |
| $x_{3}$ (Household size) | 867 | 0.000 | 2.303 | 1.176 | 0.399 |
| $x_{4}$ (Household income) | 867 | 0.000 | 1.609 | 0.861 | 0.438 |
| $x_{5}$ (Number of air conditioning units) | 867 | 0.000 | 1.792 | 1.020 | 0.437 |
| $x_{6}$ (Number of other household  appliances) | 867 | 0.000 | 3.367 | 2.518 | 0.297 |
| Number of valid cases | 867 |  |  |  |  |

**Supplementary table 3.**

**Table 3** Variance inflation factor of variables

| Variable | VIF | 1/VIF |
| --- | --- | --- |
| $x_{1}$ | 1.360 | 0.733 |
| $x_{5}$ | 1.350 | 0.740 |
| $x_{4}$ | 1.270 | 0.785 |
| $x_{6}$ | 1.260 | 0.791 |
| $x_{3}$ | 1.200 | 0.834 |
| $x_{2}$ | 1.060 | 0.943 |
| BESD | 1.020 | 0.978 |
