# Supplementary material for: Dilution effect of the building area on energy intensity in urban residential buildings
Source: Nat Commun. 2019 Oct 30;10:4944. doi: 10.1038/s41467-019-12852-9 (PMC6821746; doi:10.1038/s41467-019-12852-9)
Supplement: Supplementary file 2 — Description of Additional Supplementary Files [file 41467_2019_12852_MOESM2_ESM.pdf]

**Description of Additional Supplementary Files**

File Name: Supplementary Data 1
Description: The data for the physical characteristics of the residential buildings, the household electricity consumption and variable values, including building area per household, number of floors, household size, household income, number of air conditioning units, and number of other appliances are shown in Supplementary Data 1.

File Name: Supplementary Data 2
Description: The data for the PSM (Propensity Score Matching) model is shown in Supplementary Data 2.

File Name: Supplementary 3
Description: The source data for the fig. 3 is listed in Supplementary Data 3 and Supplementary Data 4.

File Name: Supplementary 4
Description: The source data for the fig. 3 is listed in Supplementary Data 3 and Supplementary Data 4.

Files Name: Supplementary Data 5
Description: The source data for the fig. 4 is listed in Supplementary Data 5.

File Name: Supplementary Data 6
Description: The "dilution effect" of the building area per household on the URBEC per square meter and the overestimation of the BESD effectiveness in rapidly urbanized areas are tested by multivariate linear regression model, which was conducted by stata software. The source code is the shown in supplementary data 6.
