# Supplementary material for: Dilution effect of the building area on energy intensity in urban residential buildings
Source: Nat Commun. 2019 Oct 30;10:4944. doi: 10.1038/s41467-019-12852-9 (PMC6821746; doi:10.1038/s41467-019-12852-9)
Supplement: Supplementary file 8 — Supplementary Data 6 [file 41467_2019_12852_MOESM8_ESM.pdf]

```
tabstat   y1 y2 d x1   x2 x3 x4 x5 x6 ,stats(n mean median sd min max) f(%10.4f)

pwcorr_a y1 y2 d x1   x2 x3 x4 x5 x6 , star1(0.01) star5(0.05) star10(0.1)


reg y1 d x1   x2 x3 x4 x5 x6
vif



reg y1 x1 x2 x3 x4 x5 x6
est store r1
gen dx1=d*x1
reg y1 d x1 dx1 x2 x3 x4 x5 x6
est store r2
esttab   r1 r2   , r2 ar2 compress nogap star(* 0.1 ** 0.05 *** 0.01)   b(%6.3f) brackets t

reg y2 x1 x2 x3 x4 x5 x6
est store r1
reg y2 d x1 dx1 x2 x3 x4 x5 x6
est store r2
esttab   r1 r2   , r2 ar2 compress nogap star(* 0.1 ** 0.05 *** 0.01)   b(%6.3f) brackets t


reg y1 x1 x2 x3 x4 x5 x6
est store r1
reg y1 d x1 dx1 x2 x3 x4 x5 x6
est store r2
reg y2 x1 x2 x3 x4 x5 x6
est store r3
reg y2 d x1 dx1 x2 x3 x4 x5 x6
est store r4
esttab   r1 r2 r3 r4   , r2 ar2 compress nogap star(* 0.1 ** 0.05 *** 0.01)   b(%6.3f) brackets t
```
